# A retrospective, longitudinal cohort study of trends and risk factors for preterm birth in the Northern Territory, Australia

**DOI:** 10.1186/s12884-023-06164-6

**Published:** 2024-01-05

**Authors:** Kiarna Brown, Carina Cotaru, Michael Binks

**Affiliations:** 1 Menzies School of Health Research, Royal Darwin Hospital, Building 58, John Matthews Building, Tiwi, NT 0810 Australia; 2 Department of Obstetrics and Gynaecology, Royal Darwin Hospital, Darwin, NT Australia

**Keywords:** Preterm birth, Trends, Pregnancy, Aboriginal, First nations, Northern Territory

## Abstract

**Background:**

Preterm birth (PTB) is the single most important cause of perinatal mortality and morbidity in high income countries. In Australia, 8.6% of babies are born preterm but substantial variability exists between States and Territories. Previous reports suggest PTB rates are highest in the Northern Territory (NT), but comprehensive analysis of trends and risk factors are lacking in this region. The objective of this study was to characterise temporal trends in PTB among First Nations and non-First Nations mothers in the Top End of the NT over a 10-year period and to identify perinatal factors associated with the risk of PTB.

**Methods:**

This was a retrospective population-based cohort study of all births in the Top End of the NT over the 10-year period from January 1st, 2008, to December 31st, 2017. We described maternal characteristics, obstetric complications, birth characteristics and annual trends in PTB. The association between the characteristics and the risk of PTB was determined using univariate and multivariate generalised linear models producing crude risk ratios (cRR) and adjusted risk ratios (aRR). Data were analysed overall, in First Nations and non-First Nations women.

**Results:**

During the decade ending in 2017, annual rates of PTB in the Top End of the NT remained consistently close to 10% of all live births. However, First Nations women experienced more than twice the risk of PTB (16%) compared to other women (7%). Leading risk factors for PTB among First Nations women as compared to other women included premature rupture of membranes (RR 12.33; 95% CI 11.78, 12.90), multiple pregnancy (RR 7.24; 95% CI 6.68, 7.83), antepartum haemorrhage (RR 4.36; 95% CI 3.93, 4.84) and pre-existing diabetes (RR 4.18; 95% CI 3.67, 4.76).

**Conclusions:**

First Nations women experience some of the highest PTB rates globally. Addressing specific pregnancy complications provides avenues for intervention, but the story is complex and deeper exploration is warranted. A holistic approach that also acknowledges the influence of socio-demographic influences, such as remote dwelling and disadvantage on disease burden, will be required to improve perinatal outcomes.

**Supplementary Information:**

The online version contains supplementary material available at 10.1186/s12884-023-06164-6.

## Background

Preterm birth (PTB), defined as birth between 20 and 37 completed weeks of pregnancy, is the single most important cause of perinatal mortality and morbidity in high income countries [1]. In Australia, data from 2020 demonstrate that 8.6% of babies were born preterm [2]. This translates to approximately 26,000 babies being born preterm annually, with the majority born between 32 and 36 completed weeks.

The highest burden of PTB occurs among socio-economically disadvantaged groups in low income countries [3]. Nationally during 2016 to 2018, the PTB rate among babies born to Australian First Nations women (13.8%) was nearly 1.7 times higher than among babies born to non-First Nations mothers (8.4%) [4]. The most important risk factors associated with PTB among First Nations women during this time period were antepartum haemorrhage, low rates of attending antenatal care, pre-existing diabetes mellitus, hypertension in pregnancy, being underweight and smoking during pregnancy [4].

There is also variability in the rate of PTB between Australian States and Territories. PTB rates are highest in the Northern Territory (NT) and have increased over time. The NT PTB rate was reported at 10.6% in 2010 and 11.4% in 2020, but recent temporal trends of PTB birth in the Top End of the NT have not been comprehensively studied [2]. The PTB rate among a cohort of First Nations infants born in the Royal Darwin Hospital from 1987 to 1990 was 7.4% [5]. Risk factors associated with an increased risk of PTB were gestational hypertension, prolonged ruptured membranes and smoking more than half a packet of cigarettes per day. Following that, Kildea et al. described risk factors for PTB in pregnant women in two large remote NT communities from 2004 through to 2011 spanning the introduction of a new model of antenatal care [6]. The new model, a culturally enhanced Midwifery Group Practice (MGP) consisting of midwives, Strong Women Workers and Aboriginal Health Workers working in a team in the regional hospital, provided continuity of midwifery and culturally sensitive care for women any time they came to the regional centre during pregnancy, the intrapartum period and the postnatal period. The PTB rate was 19.4%, with a non-significant reduction following introduction of the new model (20.6% vs 17.7%). Risk factors for PTB were teenage motherhood, previous PTB, inadequate antenatal visits, hypertension in pregnancy and antepartum haemorrhage.

Several other NT studies have also investigated specific factors and their impact on birth outcomes, including gestational age at birth. Such studies have examined the impact of sexually transmitted infections, vaccinations during pregnancy and young maternal age, none of which were significantly associated with PTB risk [7–10]. One study showed a higher rate of PTB in those with (versus without) rheumatic heart disease, whereas two studies found lower rates of PTB in those receiving enhanced models of pregnancy care [11–13].

Current evidence suggests high rates of PTB persist in First Nations women despite the introduction of preventative strategies that have proven successful in non-First Nations pregnancies, but the availability of enhanced antenatal care models have proven successful among First Nations women [14, 15].

The higher burden of PTB in First Nations’ women is a complex interplay between clinical risks and the cultural and socio-economic inequality which First Nations’ people face. According to the Australian Institute of Health and Welfare Aboriginal and Torres Strait Islander Health Performance Framework 2023 Summary Report, 34% of the total health gap between First Nations’ and non-First Nations’ Australians is due to the social determinants of health and only 19% is due to individual persons’ factors [16]. Racism has also been shown to negatively impact the health of those experiencing it. In a recent systematic review on the impact of racism on Australian First Nations’ people’s health, several outcomes such as increased BMI and smoking, associated with an increased risk of PTB, were shown to be associated with racism [17].

To best guide future research, prevention strategies and allocation of resources, the objective of this study was to characterise temporal PTB trends among First Nations and non-First Nations women living in the Top End of the NT from 2008 to 2017 and to identify key factors associated with the risk of PTB.

## Methods

### Study design and population

This was a retrospective population-based cohort study of all births in the Top End of the NT (> 20 weeks) over the 10-year period from January 1, 2008, to December 31, 2017. The NT of Australia is large (1.4 million km^2^) and sparsely populated (0.16 people per km^2^) [18]. Darwin, on the north coast is the largest city. The northernmost (subtropical) region is known as the Top End and includes Darwin urban, Darwin rural, East Arnhem and the Katherine health districts. As of June 2020, the estimated resident population of the Top End was 200,523 (approximately 82% of the total NT population). Just over a quarter (26%) of residents are First Nations’ Australians. The majority (66%) of First Nations residents live outside Darwin urban area [19]. Three public hospitals within the region provide maternity care. A single private hospital located in Darwin operates independently.

### Data source

Data were sourced from a single dataset, the NT perinatal data set or ‘NT Midwives’ Collection’, custodied by the Child and Youth Development Research Partnership. The NT perinatal dataset contains demographic and clinical information documented by the attending midwife or clinician for all NT pregnancies, including births in public and private hospitals, planned home births, births in unattended locations such as community health centres and other non-hospital births. All births of at least 20 weeks gestation or with a birth weight of at least 400 g are included. Gestational age at birth was calculated from the mother’s recorded Estimated Date of Confinement (EDC). This is based on either the mother’s last normal menstrual period or the earliest foetal biometry at ultrasound.

First Nations status of mothers was recorded by the midwives at birth. Perinatal data collection was validated against a Client Master Index as well as public hospital data and the First Nations’ status of mothers is considered highly reliable. In the Northern Territory, First Nations’ status in health records has been estimated to have 98% consistency between electronic patient records and self-report during interview [20]. This is exceptional when compared to other Australian data.

Measures of advantage and disadvantage were assessed using the Statistics Socio-Economic Indexes for Areas (SEIFA) metric. SEIFA divides areas into deciles based on people’s access to material and social resources determined through national census data on income, education, employment, occupation, housing and family structure. In this study we compared decile 1 (the most disadvantaged) with the remaining deciles combined.

### Analysis

Our unit of analyses was pregnancy. PTB was defined as any birth occurring less than 37 weeks gestational age (GA) with sub classifications 33–36 + 6 weeks, 28–32 + 6 weeks and 20–27 + 6 weeks, in line with previously published Australian data describing PTB trends [21]. The prevalence of maternal characteristics, obstetric complications, birth characteristics and PTB were described overall and among First Nations’ and non-First Nations’ women with between group differences, using univariate risk ratios (RR) and 95% confidence intervals (95%CI). Population specific differences in PTB were evaluated using univariate and multivariate analyses producing crude RR (cRR) and adjusted RR (aRR). A generalised linear model (log-binomial regression with robust error clustering on women’s identifier) was used for the multivariate analysis with covariables selected based on both clinical logic and univariate regression outcomes (predictors with 95% CIs that excluded 1). Effects of perinatal and other characteristics on PTB risk were also evaluated separately within each population group to highlight any differential effects. Annual PTB prevalences were plotted graphically with trends assessed using log-binomial regression. Annual gestational ages were presented graphically with trends assessed using both a nonparametric (K-sample) equality of medians test. All multivariate models were adjusted for secular trends. There was no imputation for missing data. All analyses were conducted using Stata software version 17.

## Results

**Fig. 1:**
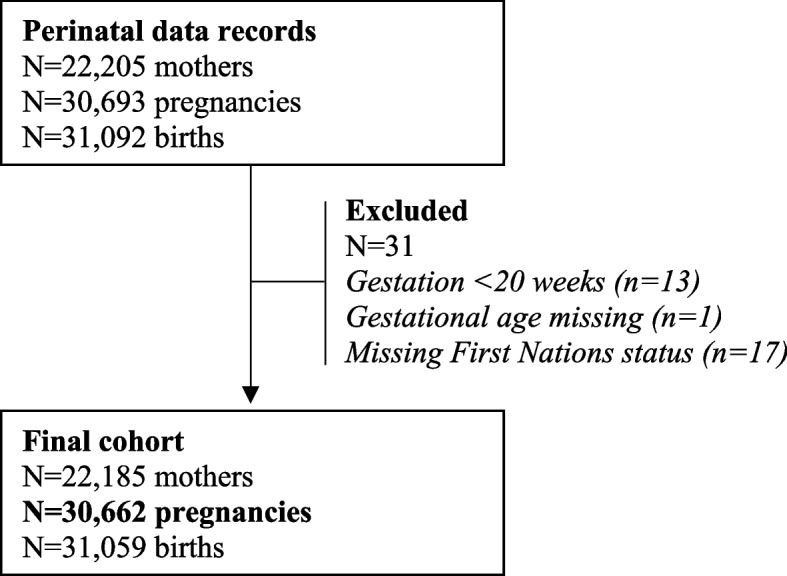
Population based preterm birth cohort

In the Top End during the study period 22,205 women had 30,693 pregnancies (Fig. 1). Thirty-one births were excluded because they occurred prior to 20 weeks gestation or key data were missing. The final cohort comprised 30,662 pregnancies; 9,153 (29.9%) were First Nations women and 9,466 (30.9% were first time women (Table 1).

**Table 1:** Perinatal characteristics in the Top End of the Northern Territory (2008–2017): first nations and non first nations

	**All Pregnancies** *N* = 30662 (100%)	**First Nations** *N* = 9153 (29.9%)	**Non-First Nations** *N* = 21509 (70.1%)	**Crude Risk Ratio (95% CI)**
**General characteristics, n (%)**				
First pregnancy	9466/30654 (30.9)	2483 (27.1)	7065/21501 (32.9)	0.83 (0.80,0.87)
Maternal age at birth (years)				
≤ *19*	2281 (7.4)	1735 (19.0)	546 (2.5)	7.45 (6.80,8.20)
*20–29*	14681 (47.9)	5264 (57.5)	9417 (43.7)	1.31 (1.28,1.34)
*30–39*	12745 (41.6)	2024 (22.1)	10721 (49.8)	0.44 (0.43,0.46)
≥ *40*	955 (3.1)	130 (1.4)	825 (3.8)	0.37 (0.31,0.44)
Antenatal care in 1^st^ trimester	23156/30323 (76.4)	5018/8960 (56.0)	18138/21363 (84.9)	0.66 (0.65,0.67)
Any smoking during pregnancy	6714/28716 (23.4)	4609/8282 (55.7)	2105/20434 (10.3)	5.40 (5.16,5.65)
Any alcohol during pregnancy	1369/28624 (4.8)	781/8017 (9.7)	588/20607 (2.9)	3.41 (3.08,3.79)
IRSAD decile 1 (most disadvantaged)	6428/30635 (21.0)	5335/9142 (58.4)	1093/21493 (5.1)	11.48 (10.80,12.19)
Remote dwelling (ARIA +)	9909/30635 (32.3)	6673/9142 (73.0)	3236/21493 (15.1)	4.85 (4.69,5.02)
**Pre-existing risk factors, n (%)**				
Anaemia	1203 (3.9)	868 (9.5)	335 (1.6)	6.09 (5.38,6.89)
Diabetes	383 (1.2)	286 (3.1)	97 (0.5)	6.93 (5.51,8.71)
Hypertension	228 (0.7)	115 (1.3)	113 (0.5)	2.39 (1.85,3.10)
Cardiac disease	702 (2.3)	589 (6.4)	113 (0.5)	12.25 (10.03,14.96)
Renal disease	276 (0.9)	223 (2.4)	53 (0.2)	9.88 (7.34,13.33)
**Obstetric complications, n (%)**				
Gestational anaemia	1660 (5.4)	1069 (11.7)	591 (2.7)	4.25 (3.86,4.69)
Gestational diabetes	3290 (10.7)	1199 (13.1)	2091 (9.7)	1.35 (1.26,1.44)
Pre-eclampsia	922 (3.0)	339 (3.7)	583 (2.7)	1.37 (1.20,1.56)
Antepartum haemorrhage	611 (2.0)	173 (1.9)	438 (2.0)	0.93 (0.78,1.10)
Intrauterine growth restriction	880 (2.9)	497 (5.4)	383 (1.8)	3.05 (2.67,3.48)
Urinary tract infection	798 (2.6)	624 (6.8)	174 (0.8)	8.43 (7.14,9.95)
Premature rupture of membranes	832 (2.7)	427 (4.7)	405 (1.9)	2.48 (2.17,2.83)
Multiple pregnancy	418 (1.4)	128 (1.4)	290 (1.3)	1.04 (0.84,1.28)
**Birth characteristics, n (%)**				
Public hospital birth	22455 (73.2)	8540 (93.3)	13915 (64.7)	1.44 (1.43,1.46)
Private hospital birth	6540 (21.3)	120 (1.3)	6420 (29.8)	0.04 (0.04,0.05)
Born outside hospital	1667 (5.4)	493 (5.4)	1174 (5.5)	0.99 (0.89,1.09)
Vaginal birth	20817 (67.9)	6477 (70.8)	14340 (66.7)	1.06 (1.04,1.08)
Induced birth	7943 (25.9)	2040 (22.3)	5903 (27.4)	0.81 (0.78,0.85)
Elective Caesarean birth	4763 (15.5)	1009 (11.0)	3754 (17.5)	0.63 (0.59,0.67)
Emergency Caesarean birth	5082 (16.6)	1667 (18.2)	3415 (15.9)	1.15 (1.09,1.21)

*CI* confidence interval

Women attended antenatal care during the first trimester in 23,156 (76.4%) pregnancies. Pre-existing maternal medical risk factors each occurred in less than 4% of women. Gestational diabetes was diagnosed in 3,290 (10.7%) pregnancies. Complications including pre-eclampsia, antepartum haemorrhage, intrauterine growth restriction, urinary tract infection and premature rupture of membranes each occurred in less than 4% of all pregnancies. Multiple pregnancies accounted for 1.4% of the cohort. Most births (73.2%) happened in public hospitals. Twenty-six per cent of pregnancies had birth induced and 67.9% of women had a vaginal birth. The caesarean section rate was 32.1%, including both 4,763 elective (15.5%) and 5,082 emergency (16.6%) caesareans (Table 1).

First Nations women were more likely to live remotely (cRR 4.85; 95% CI 4.69, 5.02), be socially disadvantaged (RR 11.48; 95% CI 10.80, 12.19), smoke during pregnancy (cRR 5.40; 95% CI 5.16, 5.65) and have pre-existing maternal medical risk factors including renal disease (cRR 9.88; 95% CI 7.34, 13.33), diabetes (cRR 6.93; 95% CI 5.51, 8.71), anaemia (cRR 6.09; 95% CI 5.38, 6.89) and hypertension (cRR 2.39; 95% CI 1.85, 3.10). First Nations women had a substantially higher burden of cardiac disease (cRR 12.25; 95% CI 10.03, 14.96).

In pregnancy, First Nations’ women were more likely to experience premature pre-labour rupture of membranes (PPROM; cRR 2.48; 95% CI 2.17, 2.83) and intrauterine growth restriction (cRR 3.05; 95% CI 2.67, 3.48). First Nations’ women were 4 times (cRR 4.25; 95% CI 3.86, 4.69) more likely to develop anaemia and 8 times (cRR 8.43; 95% CI 7.14, 9.95) more likely to develop urinary tract infections during pregnancy. Gestational diabetes, pre-eclampsia and antepartum haemorrhage were more similar in prevalence among First Nations’ and non-First Nations’ pregnancies (Table 1).

**Fig. 2:**
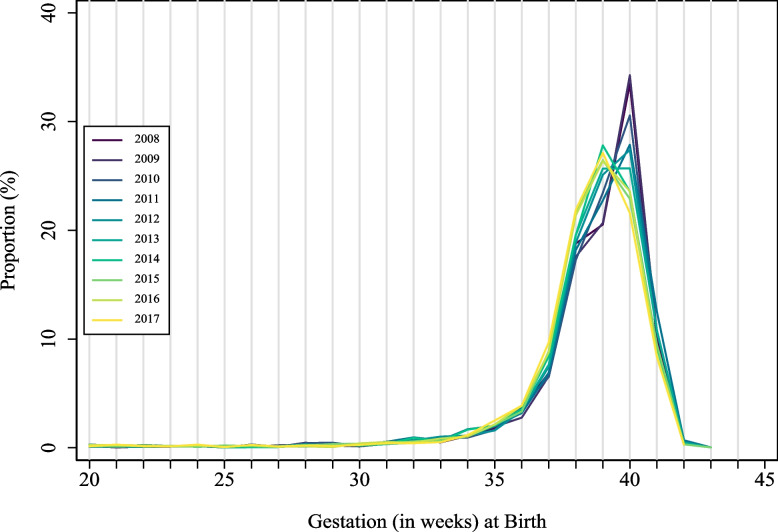
Frequency distribution of gestational age at birth (%) by calendar year: all Top End (Northern Territory) births

**Fig. 3:**
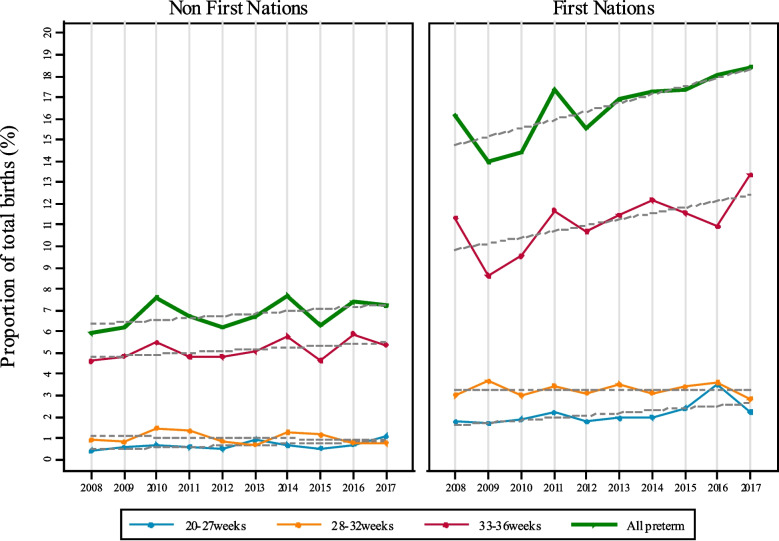
Annual preterm birth prevalence in the Top End of the Northern Territory (2008–2017): first nations and non first nations

The overall prevalence of PTB was 9.7% (Table 2) with little temporal variation (Supplementary Table 2) despite a significant shift in median GA at birth (from 40 to 39 weeks) by calendar year (*p* < 0.001; Fig. 2). Compared to other women, First Nations’ women had a higher risk of PTB overall (16.5% versus 6.8%; aRR 1.52; 95% CI 1.36, 1.70) and across all preterm gestational age categories (Table 2, Fig. 3). Further, there was a positive trend in PTB’s among First Nations’ women by calendar year (RR 1.02; 95% CI 1.01, 1.04, Fig. 3, Supplementary Table 2), that was predominately driven by the increase in births in the 33-to-36^+6^-week gestational age group.

**Table 2:** Risk of preterm birth in the Top End of the Northern Territory (2008–2017): first nations and non first nations

	**All Pregnancies** *N* = 30,662	**First Nations** *N* = 9153	**Non-First Nations** *N* = 21,509	**Risk Ratio (95% CI)**	**Adjusted Risk Ratio (95% CI)**
**Preterm Birth, n (%)**					
Any, < 37 weeks	2970 (9.7)	1506 (16.5)	1464 (6.8)	2.42 (2.26,2.59)	1.52 (1.36,1.70)
*33–36 weeks*	2119 (6.9)	1013 (11.1)	1106 (5.1)	2.15 (1.98,2.34)	1.39 (1.22,1.59)
*28–32 weeks*	515 (1.7)	300 (3.3)	215 (1.0)	3.28 (2.76,3.90)	1.71 (1.26,2.32)
*20–27 weeks*	336 (1.1)	193 (2.1)	143 (0.7)	3.17 (2.56,3.93)	1.96 (1.37,2.82)

Adjusted risk ratios were generated using a generalised linear model (log-binomial with robust error) with adjustment for secular trends. Model included the following covariates: year of birth, first pregnancy, maternal age (category) at birth, received antecare in first trimester, previous preterm birth, documented smoking in pregnancy (missing *n* = 1946), documented alcohol use in pregnancy (missing *n* = 2038), Index of Relative Socio-economic Advantage and Disadvantage decile 1 (most disadvangtaged; missing, *n* = 27), remoteness dwelling (ARIA + ; missing, *n* = 27), pre-existing anaemia, pre-existing diabetes, pre-existing hypertension, pre-existing cardiac disease, pre-existing renal disease, gestational anaemia, gestational diabetes, preeclampsia, antepartum haemorrhage, intrauterine growth restriction, urinary tract infection in pregnancy, premature rupture of membranes, induced birth, elective Caesar

*CI* confidence interval

When assessed independently, smoking during pregnancy was associated with an increased risk of PTB (cRR 1.90; 95% CI 1.77, 2.05). However, when adjusted for other factors, the association was lower (aRR 1.24; 95% CI 1.13, 1.37). The same was true for socio-economic disadvantage. Higher rates of PTB were seen in those classified as most disadvantaged (Index of Relative Socio-economic Advantage and Disadvantage decile 1) (cRR 1.98; CI 95% 1.84, 2.12 Supplementary Table 1).

Though many factors were more prevalent among First Nations’ women, when present, their magnitude of association with PTB was similar for both First Nations’ and non-First Nations’ pregnancies (Supplementary Table 1). The largest associations among First Nations’ and non-First Nations’ pregnancies were for PPROM (aRR 8.98; 95% CI 8.12, 9.94; aRR 20.61; 95% CI 18.46, 23.08), multiple pregnancy (aRR 5.50; 95% CI 4.42, 6.83; aRR 9.44; 95% CI 7.64, 11.66), preeclampsia (aRR 3.56; 95% CI 2.93, 4.33; aRR 6.28; 95% CI 5.23, 7.55), antepartum haemorrhage (aRR 3.19; 95% CI 2.45, 4.17; aRR 6.06; 95% CI 4.94, 7.44), pre-existing diabetes (aRR 2.69; 95% CI 2.11, 3.43; aRR 4.67; 95% CI 3.05, 7.14) and intrauterine growth restriction (aRR 2.25; 95% CI 1.90, 2.67; aRR 3.36; 95% CI 2.61, 4.33) (Supplementary Table 1).

In our study, PPROM occurred in 4.7% of First Nations’ pregnancies and 93% of women who had the condition gave birth preterm. Conversely, 1.9% of non-First Nations pregnancies were complicated by PPROM and 89% gave birth preterm (Supplementary Table 1).

## Discussion

During the decade ending in 2017, the annual prevalence of PTB in the Top End of the NT remained consistently close to 10% of total live births which is considerably higher than reported nationally (8.6%) [2]. Of concern, First Nations’ women had around twice the risk of PTB compared to other women and the gap appeared to be widening. The PTB prevalence reached 18% in First Nations’ women in 2016–2017 compared with 6–8% in non-First Nations’ women.

Globally PTB is a critical concern, representing the leading cause of both neonatal and childhood mortality [22]. The World Health Organisation estimated the global average rate of PTB in 2010 to be 11.1%. Across countries, PTB rates ranged from 5 to 18%, with the highest rates seen in Africa and southern Asia [23]. Our study highlights that the unique geographical, physical, and social environment of the Top End gives way to PTB rates in our First Nations’ population that are amongst the highest in the world.

It is important to note that the risk factors for PTB are the same for First Nations and non-First Nations pregnancies, but the rate at which the risk factors occur in First Nations pregnancies is often much higher. Smoking represents an important modifiable contributing factor with rates in our study much higher in First Nations women compared with non-First nations women (56% vs 10%, Table 1). There has been a small improvement in addressing smoking cessation during pregnancy in First Nations women across Australia, with latest data reporting a decrease from 48% in 2011 to 43% in 2020 [4]. More must be done to promote smoking cessation in First Nations women of the Top End.

Premature rupture of membranes is common, particularly in Top End First Nations women and strongly associated with PTB. The pathophysiology for PPROM remains largely unexplained. While a wide array of mechanisms cause PPROM, it is commonly associated with infection [24]. Ascending infection from the lower genital tract to the amniotic fluid is considered the most frequent route of infection [25]. A comprehensive understanding of the vaginal microbiome as well as genetic factors that dictate an inflammatory response is required to understand why some women develop infection leading to PPROM, but most do not. Currently little is known about the vaginal microbial composition in First Nations women. Further research is underway and will hope to add vital and so far unknown information to better understand this condition in First Nations women of the Top End [26].

Since 1987 there have been two studies assessing the risk factors for PTB in the Top End [5, 6]. Both studies as well as our study have identified hypertensive disorders in pregnancy as a significant risk factor for PTB. Prediction and prevention of pregnancy hypertension have been explored in recent years. Prediction tools have included uterine artery Doppler studies and measurement of angiogenic factors [27]. These tools are yet to be trialled and adopted in our population. Prevention strategies have included prescription of calcium and aspirin, but the effectiveness of these have not been tested in this population [28]. While no treatment to date can reliably prevent pregnancy hypertension in all women, this remains an area for further development in this population [29].

Enhanced screening, diagnosis and treatment of pre-existing medical disorders could have an important role in reducing PTB rates. Our study demonstrated a strong association between pre-existing diabetes and PTB and epidemiological studies have shown a 10-fold increase in pre-existing diabetes among pregnant First Nations women over the last 30 years [30]. Combined, these suggest that enhanced diabetes prevention and management strategies could reduce preterm births to some extent.

Several perinatal factors did not appear to be associated with PTB. For example, gestational diabetes mellitus had little impact on PTB rates for both First Nations women and non-First Nations women. Urinary tract infection, pre-existing anaemia and gestational anaemia did not appear to be associated with PTB.

Many social determinants of health influence rates of PTB. In our study, remote dwelling was associated with an increased PTB risk, but the underlying factors are unclear. Many important social determinants were not captured in our data such as educational attainment, employment, and access to adequate housing. Similarly, we did not have data on intimate partner violence (IPV). A meta-analysis indicated that women who experience IPV during pregnancy are at increased risk of having adverse birth outcomes, including PTB [31], and hospitalisation rates due to assault were increased in Australian First Nations women [16]. This is another, largely ill-addressed matter affecting First Nations women of the Top End. Very little evidence exists about the prevalence of IPV in this population and its impact on pregnancy outcomes. There is an opportunity to better address this issue. Training to support health care workers to identify and aid in managing IPV is essential, as are resources to support victims. Both should be explored.

Iatrogenic PTB occurred more frequently in First Nations pregnancies. We note that induction of labour did not independently increase PTB and that the higher induction rates in First Nations women were reflective of the higher rates of risk factors contributing to PTB. However, our data did not include the indications for induction. A more detailed analysis of induction could potentially identify avenues for reducing medically indicated preterm birth.

A challenge for this type of study is access to up-to-date data from the NT. Data were sourced from the NT Midwives’ perinatal data set that underwent a rigorous process of data cleansing prior to publication. This process can delay access to contemporaneous data. Further, we have not captured data on lifestyle and other important sociodemographic factors that may contribute to PTB, nor have we assessed PTB rates and contributing perinatal risk factors in other vulnerable populations such as migrant and refugee women.

The aetiology of PTB is most likely multifaceted rather than due to a single independent factor. While it may be possible to address single medical and obstetric conditions, treating these factors separately is unlikely to reach a significant reduction in overall PTB rates. A holistic approach, addressing key socio-demographic factors is key.

There was a slight reduction in PTB demonstrated in First Nations women in 2009–2010. This coincided with a redesign of maternity care in mid-2009. The new clinical care focused on remote-dwelling First Nations women of all risk profiles. The objective was to increase continuity of care and co-ordination of care through a midwifery group practice. This supports previous evidence that has shown significantly improved maternal and infant outcomes with redesign of care where First Nations leadership and holistic, culturally safe care was central [15].

Racism, whether occurring through passive inaction or deliberate action, has major adverse effects on the health of First Nations Australians and substantially hinders access to effective health care in mainstream health care [32]. Hence, it is crucial that health care for First Nations people is comprised of First Nations leadership and workforce, addressing socio-economic inequalities, free of racism.

## Strengths and limitations

The major strength of this study is the size of the study population, with 10 years of data and inclusion of over 99% of pregnancies. The large sample size and duration of the study allowed us to gain meaningful insight in the temporal trends in PTB both over time and across multiple gestational age sub-categories. The large cohort allowed us to demonstrate statistically significant associated factors for PTB. Additionally, the large proportion of First Nations women allowed valuable insights and meaningful comparisons between First Nations and non-First Nations women. We used the most reliable data source.

Using a retrospective population-based cohort study design based on administrative data is appropriate, but limits comparisons with control groups and unrecognised confounders may influence the results. Important pre-existing maternal factors that could contribute to PTB such as a previous history of PTB was not recorded in the dataset and therefore their contribution could not be analysed. Although we assessed major contributing factors, we could not demonstrate the impact of each of these over time. Likewise, we could not demonstrate the interactions between factors and the impact on PTB, for example in women with pre-existing diabetes who also developed hypertensive disorders during pregnancy. We were unable to investigate the indications for induced preterm birth as this was not recorded. We did not compare PTB rates between public and private hospital settings, acknowledging that very few First Nations women gave birth in the private hospital in this population.

## Conclusions

This comprehensive longitudinal cohort study clearly demonstrates the trends and high burden of PTB in the Top End of the NT. Alarmingly, First Nations women experienced some of the highest PTB rates globally. While PTB risk factors were the same for both First Nations and non-First Nations pregnancies, they were much more common in First Nations women. Smoke exposure, socioeconomic factors, chronic diseases and pregnancy complications are avenues for intervention, but the story is complex and deeper exploration is warranted. Further prospective cohort studies are necessary to provide clearer understanding of PTB risk and to evaluate previously unexplored issues such as intimate partner violence and indications for iatrogenic PTB.

A call for action has previously been declared by Hickey et al., arguing for the urgent need for adequately funded Indigenous-led solutions to perinatal health inequities for Indigenous families in well-resourced settler-colonial countries and we, clinicians and researchers from the Top End of the NT, support this call [33].

### Supplementary Information

**Additional file 1: Supplementary Table 1.** Risk of preterm birth among First Nations and non-First Nations women (2008-2017): by perinatal characteristics.

**Additional file 2: Supplementary Table 2.** Annual preterm birth prevalence (2008-2017) including subcategories: A: All pregnancies, B: First Nations and C: Non First Nations.

## Data Availability

The authors do not have permission to share patient-level data extracted from the NT Midwives perinatal data set or ‘NT Midwives’ Collection’. Data can only be made available to researchers who apply to the Human Research Ethic Committee of the Northern Territory Department of Health and Menzies School of Health Research (Ethics - Menzies) and the Child and Youth Development Research Partnership repository (Child and Youth Development Research Partnership CYDRP 2017-2024 - Menzies).

## Abbreviations

PTB: Preterm birth
NT: Northern Territory
MGP: Midwifery group practice
GA: Gestational age
PPROM: Premature prelabour rupture of membranes
IPV: Intimate partner violence
