# Supplementary material for: A retrospective, longitudinal cohort study of trends and risk factors for preterm birth in the Northern Territory, Australia
Source: BMC Pregnancy Childbirth. 2024 Jan 5;24:33. doi: 10.1186/s12884-023-06164-6 (PMC10768210; doi:10.1186/s12884-023-06164-6)
Supplement: Supplementary file 1 — Additional file 1: Supplementary Table 1. Risk of preterm birth among First Nations and non-First Nations women (2008-2017): by perinatal characteristics. [file 12884_2023_6164_MOESM1_ESM.docx]

**Supplementary Table 1.** Risk of preterm birth among First Nations and non-First Nations women (2008-2017): by perinatal characteristics

|  | **All Pregnancies (N=30662)** | **First Nations (N=9153)** | **Non-First Nations (N=21509)** |
| --- | --- | --- | --- |

|  |  | **Preterm, n (%)** | **RR**  **(95% CI)** | **aRR**  **(95% CI)** |  | **Preterm, n (%)** | **RR**  **(95% CI)** | **aRR**  **(95% CI)** |  | **Preterm, n (%)** | **RR**  **(95% CI)** | **aRR**  **(95% CI)** |
| --- | --- | --- | --- | --- | --- | --- | --- | --- | --- | --- | --- | --- |
| **General characteristics** |  |  |  |  |  |  |  |  |  |  |  |  |
| First pregnancy | Yes, n=9466 | 852 (9) | 0.90 (0.83, 0.97) | 0.95 (0.86,1.04) | Yes, n=2483 | 357 (14) | 0.83 (0.75,0.93) | 0.81 (0.69,0.95) | Yes, n=6983 | 495 (7) | 1.06 (0.96,1.18) | 0.98 (0.87,1.10) |
|  | No, n=21188 | 2118 (10) |  |  | No, n=6670 | 1149 (17) |  |  | No, n=14518 | 969 (7) |  |  |
| Maternal age at birth (yr) |  |  |  |  |  |  |  |  |  |  |  |  |
| ≤19 | Yes, n=2281 | 303 (13) | Ref (1.00) | Ref (1.00) | Yes, n=1735 | 259 (15) | Ref (1.00) | Ref (1.00) | Yes, n=546 | 44 (8) | Ref (1.00) | Ref (1.00) |
| 20-29 | Yes, n=14681 | 1446 (10) | 0.74 (0.66,0.83) | 0.96 (0.82,1.12) | Yes, n=5264 | 844 (16) | 1.07 (0.94,1.22) | 0.94 (0.79,1.13) | Yes, n=9417 | 602 (6) | 0.79 (0.59,1.06) | 0.87 (0.62,1.24) |
| 30-39 | Yes, n=12745 | 1124 (9) | 0.66 (0.59,0.75) | 1.05 (0.89,1.24) | Yes, n=2024 | 374 (18) | 1.24 (1.07,1.43) | 0.99 (0.80,1.24) | Yes, n=10721 | 750 (7) | 0.87 (0.65,1.16) | 0.98 (0.69,1.38) |
| ≥40 | Yes, n=955 | 97 (10) | 0.76 (0.61,0.95) | 1.12 (0.86,1.46) | Yes, n=130 | 29 (22) | 1.49 (1.06,2.10) | 1.05 (0.66,1.66 | Yes, n=825 | 68 (8) | 1.02 (0.71,1.47) | 1.03 (0.67,1.58) |
| Antenatal care in 1^st^ trimester | Yes, n=23156 | 1975 (9) | 0.69 (0.64,0.75) | 0.95 (0.87,1.05) | Yes, n=5018 | 775 (15) | 0.94 (0.85,1.03) | 0.90 (0.80,1.01) | Yes, n=18138 | 1200 (7) | 0.92 (0.80,1.05) | 1.04 (0.88,1.22) |
|  | No, n=7167 | 881 (12) |  |  | No, n=3942 | 649 (16) |  |  | No, n=3225 | 232 (7) |  |  |
| Any smoking during pregnancy | Yes, n=6714 | 966 (14) | 1.90 (1.77,2.05) | 1.24 (1.13,1.37) | Yes, n=4609 | 782 (17) | 1.24 (1.12,1.37) | 1.20 (1.07,1.35) | Yes, n=2105 | 184 (9) | 1.38 (1.19,1.60) | 1.23 (1.04,1.45) |
|  | No, n=22002 | 1663 (8) |  |  | No, n=3673 | 503 (14) |  |  | No, n=18329 | 1160 (6) |  |  |
| Any alcohol during pregnancy | Yes, n=1369 | 199 (15) | 1.67 (1.46,1.91) | 1.10 (0.93,1.29) | Yes, n=781 | 151 (19) | 1.33 (1.14,1.55) | 1.06 (0.89,1.27) | Yes, n=588 | 48 (8) | 1.24 (0.94,1.64) | 1.19 (0.87,1.63) |
|  | No, n=27255 | 2371 (9) |  |  | No, n=7236 | 1055 (15) |  |  | No, n=20019 | 1316 (7) |  |  |
| IRSAD decile 1 (most disadvantaged) | Yes, n=6428 | 1021 (16) | 1.98 (1.84,2.12) | 1.03 (0.90,1.18) | Yes, n=5335 | 951 (18) | 1.23 (1.19,1.36) | 0.89 (0.77,1.03) | Yes, n=1093 | 70 (6) | 0.94 (0.74,1.18) | 1.14 (0.86,1.63) |
|  | No, n=24207 | 1943 (8) |  |  | No, n=3807 | 551 (14) |  |  | No, n=20400 | 1392 (7) |  |  |
| Remote dwelling (ARIA+) | Yes, n=9909 | 1402 (14) | 1.88 (1.75,2.01) | 0.97 (0.86,1.10) | Yes, n=6673 | 1197 (18) | 1.45 (1.29,1.63) | 1.29 (1.09,1.54) | Yes, n=3236 | 205 (6) | 0.92 (0.80,1.06) | 0.80 (0.67,0.96) |
|  | No, n=20726 | 1562 (8) |  |  | No, n=2469 | 305 (12) |  |  | No, n=18257 | 1257 (7) |  |  |
| **Pre-existing risk factors** |  |  |  |  |  |  |  |  |  |  |  |  |
| Anaemia | Yes, n=1203 | 177 (15) | 1.55 (1.35,1.79) | 0.95 (0.79,1.14) | Yes, n=868 | 163 (19) | 1.16 (1.00,1.34) | 1.02 (1.09,1.54) | Yes, n=335 | 14 (4) | 0.61 (0.36,1.02) | 0.61 (0.34,1.08) |
|  | No, n=29459 | 2793 (9) |  |  | No, n=8285 | 1343 (16) |  |  | No, n=21174 | 1450 (7) |  |  |
| Diabetes | Yes, n=383 | 149 (39) | 4.18 (3.67,4.76) | 3.17 (2.53,3.97) | Yes, n=286 | 116 (41) | 2.59 (2.23,3.00) | 2.69 (2.11,3.43) | Yes, n=97 | 33 (34) | 5.09 (3.84,6.75) | 4.67 (3.05,7.14) |
|  | No, n=30279 | 2821 (9) |  |  | No, n=8867 | 1390 (16) |  |  | No, n=21412 | 1431 (7) |  |  |
| Hypertension | Yes, n=228 | 58 (25) | 2.66 (2.12,3.33) | 1.32 (0.97,1.80) | Yes, n=115 | 41 (36) | 2.20 (1.71,2.82) | 1.32 (0.93,1.90) | Yes, n=113 | 7 (15) | 2.22 (1.43,3.45) | 1.41 (0.82,2.42) |
|  | No, n=30434 | 2912 (10) |  |  | No, n=9038 | 1465 (16) |  |  | No, n=21396 | 1447 (7) |  |  |
| Cardiac disease | Yes, n=702 | 133 (19) | 2.00 (1.71,2.34) | 1.11 (0.91,1.35) | Yes, n=589 | 119 (20) | 1.25 (1.06,1.48) | 1.12 (0.92,1.35) | Yes, n=113 | 14 (12) | 1.83 (1.12,2.99) | 1.11 (0.69,1.79) |
|  | No, n=29960 | 2837 (9) |  |  | No, n=8564 | 1387 (16) |  |  | No, n=21396 | 1450 (7) |  |  |
| Renal | Yes, n=276 | 66 (24) | 2.50 (2.02,3.10) | 1.34 (0.97,1.86) | Yes, n=223 | 60 (27) | 1.66 (1.33,2.07) | 1.27 (0.94, 1.72) | Yes, n=53 | 6 (11) | 1.67 (0.78,3.54) | 2.36 (1.05,5.32) |
|  | No, n=30386 | 2904 (10) |  |  | No, n=8930 | 1446 (16) |  |  | No, n=21456 | 1458 (7) |  |  |
| **Obstetric complications** |  |  |  |  |  |  |  |  |  |  |  |  |
| Gestational anaemia | Yes, n=1660 | 192 (12) | 1.21 (1.05,1.39) | 1.01 (0.86,1.19) | Yes, n=1069 | 160 (15) | 0.90 (0.77,1.05) | 1.01 (0.85,1.20) | Yes, n=591 | 32 (5) | 0.79 (0.56,1.11) | 0.87 (0.60,1.26) |
|  | No, n=29002 | 2778 (10) |  |  | No, n=8084 | 1346 (17) |  |  | No, n=20918 | 1432 (7) |  |  |
| Gestational diabetes | Yes, n=3290 | 356 (11) | 1.13 (1.02,1.26) | 1.25 (1.12,1.41) | Yes, n=1199 | 191 (16) | 0.96 (0.84,1.11) | 1.12 (0.95,1.32) | Yes, n=2091 | 165 (8) | 1.18 (1.00,1.38) | 1.34 (1.14,1.58) |
|  | No, n=27372 | 2614 (10) |  |  | No, n=7954 | 1315 (17) |  |  | No, n=19418 | 1299 (7) |  |  |
| Pre-eclampsia | Yes, n=922 | 301 (33) | 3.64 (3.29,4.02) | 4.98 (4.33,5.73) | Yes, n=339 | 141 (42) | 2.69 (2.35,3.07) | 3.56 (2.93,4.33) | Yes, n=583 | 160 (27) | 4.40 (3.82,5.08) | 6.28 (5.23,7.55) |
|  | No, n=29740 | 2669 (9) |  |  | No, n=8814 | 1364 (15) |  |  | No, n=20926 | 1304 (6) |  |  |
| Antepartum haemorrhage | Yes, n= | 611 (40) | 4.36 (3.93,4.84) | 4.79 (4.04,5.67) | Yes, n=173 | 104 (60) | 3.85 (3.38,4.39) | 3.19 (2.45,4.17) | Yes, n=438 | 138 (32) | 5.01 (4.32,5.80) | 6.06 (4.94,7.44) |
|  | No, n= | 2728 (9) |  |  | No, n=8980 | 1402 (16) |  |  | No, n=21071 | 1326 (6) |  |  |
| Intrauterine growth restriction | Yes, n=880 | 252 (29) | 3.14 (2.81,3.50) | 2.61 (2.25,3.04) | Yes, n=497 | 160 (32) | 2.07 (1.81,2.37) | 2.25 (1.90,2.67) | Yes, n=383 | 92 (24) | 3.70 (3.07,4.45) | 3.36 (2.61,4.33) |
|  | No, n=29782 | 2718 (9) |  |  | No, n=8656 | 1346 (16) |  |  | No, n=21126 | 1372 (6) |  |  |
| Urinary tract infection | Yes, n=798 | 130 (16) | 1.71 (1.46,2.01) | 1.12 (0.90,1.39) | Yes, n=624 | 111 (18) | 1.09 (0.91,1.30) | 1.09 (0.88,1.36) | Yes, n=174 | 19 (11) | 1.61 (1.05,2.47) | 1.04 (0.57,1.89) |
|  | No, n=29864 | 2840 (10) |  |  | No, n=8529 | 1395 (16) |  |  | No, n=21335 | 1445 (7) |  |  |
| Premature rupture of membranes | Yes, n=832 | 760 (91) | 12.33 (11.78,12.90) | 14.20 (13.11, 15.37) | Yes, n=427 | 398 (93) | 7.34 (6.91,7.80) | 8.98 (8.12,9.94) | Yes, n=405 | 362 (89) | 17.12 (16.02,18.30) | 20.61 (18.46,23.08) |
|  | No, n=29830 | 2210 (7) |  |  | No, n=8726 | 1108 (13) |  |  | No, n=21104 | 1102 (5) |  |  |
| Multiple pregnancy | Yes, n=418 | 270 (65) | 7.24 (6.68,7.83) | 7.95 (6.81,9.28) | Yes, n=128 | 95 (74) | 4.75 (4.24,5.31) | 5.50 (4.42,6.83) | Yes, n=290 | 175 (60) | 9.93 (8.92,11.06) | 9.44 (7.64,11.66) |
|  | No, n=30244 | 2700 (9) |  |  | No, n=9025 | 1411 (16) |  |  | No, n=21219 | 1289 (6) |  |  |
| **Birth characteristics** |  |  |  |  |  |  |  |  |  |  |  |  |
| Induced birth | Yes, n=7943 | 586 (7) | 0.70 (0.64,0.77) | 0.56 (0.51,0.62) | Yes, n=2040 | 295 (14) | 0.85 (0.76,0.96) | 0.66 (0.58,0.75) | Yes, n=5903 | 291 (5) | 0.66 (0.58,0.74) | 0.51 (0.44,0.59) |
|  | No, n=22719 | 2384 (10) |  |  | No, n=7113 | 1211 (17) |  |  | No, n=15606 | 1173 (8) |  |  |
| Elective Caesarean | Yes, n=4763 | 289 (6) | 0.59 (0.52,0.66) | 0.57 (0.50,0.65) | Yes, n=1009 | 87 (9) | 0.49 (0.40,0.61) | 0.45 (0.35,0.56) | Yes, n=3754 | 202 (5) | 0.76 (0.66,0.87) | 0.62 (0.53,0.73) |
|  | No, n=25899 | 2681 (10) |  |  | No, n=8144 | 1419 (17) |  |  | No, n=17755 | 1262 (7) |  |  |

Adjusted risk ratios were generated using a generalised linear model (log-binomial with robust error) with adjustment for secular trends. Models were stratified by ethnicity (First Nations and non-First Nations) and included the following covariates: year of birth, first pregnancy (missing, n=8), maternal age (category) at birth, received antenatal care in first trimester (missing, n=339), documented smoking in pregnancy (missing n=1946), documented alcohol use in pregnancy (missing n=2038), Index of Relative Socio-economic Advantage and Disadvantage decile 1 (most disadvantaged; missing, n=27), remoteness dwelling (ARIA+; missing, n=27), pre-existing anaemia, pre-existing diabetes, pre-existing hypertension, pre-existing cardiac disease, pre-existing renal disease, gestational anaemia, gestational diabetes, preeclampsia, antepartum haemorrhage, intrauterine growth restriction, urinary tract infection in pregnancy, premature rupture of membranes, induced birth, elective Caesarean. **RR**: Crude Risk Ratio. **aRR**: adjusted Risk Ratio. **CI**: confidence interval.
