# Supplementary material for: A retrospective, longitudinal cohort study of trends and risk factors for preterm birth in the Northern Territory, Australia
Source: BMC Pregnancy Childbirth. 2024 Jan 5;24:33. doi: 10.1186/s12884-023-06164-6 (PMC10768210; doi:10.1186/s12884-023-06164-6)
Supplement: Supplementary file 2 — Additional file 2: Supplementary Table 2. Annual preterm birth prevalence (2008-2017) including subcategories: A: All pregnancies, B: First Nations and C: Non First Nations. [file 12884_2023_6164_MOESM2_ESM.docx]

**Supplementary Table 2**. Annual preterm birth prevalence (2008-2017) including subcategories: A: All pregnancies, B: First Nations and C: Non First Nations.

| **A: All Pregnancies** | **2008**  **N=2960** | **2009**  **N=2990** | **2010**  **N=2937** | **2011**  **N=2992** | **2012**  **N=3161** | **2013**  **N=3181** | **2014**  **N=3140** | **2015**  **N=3142** | **2016**  **N=3132** | **2017**  **N=3027** |
| --- | --- | --- | --- | --- | --- | --- | --- | --- | --- | --- |
| **Preterm Birth, n (%)** |  |  |  |  |  |  |  |  |  |  |
| Any, <37 weeks | 273 (9.2) | 264 (8.8) | 289 (9.8) | 303 (10.1) | 286 (9.0) | 303 (9.5) | 324 (10.3) | 296 (9.4) | 323 (10.3) | 309 (10.2) |
| *33-36 weeks* | 201 (6.8) | 183 (6.1) | 200 (6.8) | 210 (7.0) | 209 (6.6) | 218 (6.9) | 236 (7.5) | 207 (6.6) | 228 (7.3) | 227 (7.5) |
| *28-32 weeks* | 47 (1.6) | 53 (1.8) | 58 (2.0) | 60 (2.0) | 49 (1.6) | 47 (1.5) | 56 (1.8) | 56 (1.8) | 49 (1.6) | 40 (1.3) |
| *20-27 weeks* | 25 (0.8) | 28 (0.9) | 31 (1.1) | 33 (1.1) | 28 (0.9) | 38 (1.2) | 32 (1.0) | 33 (1.1) | 46 (1.5) | 42 (1.4) |

| **B: First Nations** | **2008**  **N=962** | **2009**  **N=1011** | **2010**  **N=965** | **2011**  **N=958** | **2012**  **N=963** | **2013**  **N=882** | **2014**  **N=862** | **2015**  **N=881** | **2016**  **N=860** | **2017**  **N=809** |
| --- | --- | --- | --- | --- | --- | --- | --- | --- | --- | --- |
| **Preterm Birth, n(%)** |  |  |  |  |  |  |  |  |  |  |
| Any, <37 weeks | 155 (16.1) | 141 (13.9) | 139 (14.4) | 166 (17.3) | 150 (15.6) | 149 (16.9) | 146 (16.9) | 153 (17.4) | 155 (18.0) | 149 (18.4) |
| *33-36 weeks* | 109 (11.3) | 87 (8.6) | 92 (9.5) | 112 (11.7) | 103 (10.7) | 101 (11.5) | 105 (12.2) | 102 (11.6) | 94 (10.9) | 108 (13.3) |
| *28-32 weeks* | 29 (3.0) | 37 (3.6) | 29 (3.0) | 33 (3.4) | 30 (3.1) | 31 (3.5) | 27 (3.1) | 30 (3.4) | 31 (3.6) | 23 (2.8) |
| *20-27 weeks* | 7 (0.7) | 17 (1.7) | 18 (1.9) | 21 (2.2) | 17 (1.8) | 17 (1.9) | 17 (2.0) | 21 (2.4) | 30 (3.5) | 18 (2.2) |

| **C: Non-First Nations** | **2008**  **N=1998** | **2009**  **N=1979** | **2010**  **N=1972** | **2011**  **N=2034** | **2012**  **N=2198** | **2013**  **N=2299** | **2014**  **N=2278** | **2015**  **N=2261** | **2016**  **N=2272** | **2017**  **N=2218** |
| --- | --- | --- | --- | --- | --- | --- | --- | --- | --- | --- |
| **Preterm Birth, n(%)** |  |  |  |  |  |  |  |  |  |  |
| Any, <37 weeks | 118 (5.9) | 123 (6.2) | 150 (7.6) | 137 (6.7) | 136 (6.2) | 154 (6.7) | 175 (7.7) | 143 (6.3) | 168 (7.4) | 160 (7.2) |
| *33-36 weeks* | 92 (4.6) | 96 (4.9) | 108 (5.5) | 98 (4.8) | 106 (4.8) | 117 (5.1) | 131 (5.8) | 105 (4.6) | 134 (5.9) | 119 (5.4) |
| *28-32 weeks* | 18 (0.9) | 16 (0.8) | 29 (1.5) | 27 (1.3) | 19 (0.9) | 16 (0.7) | 29 (1.3) | 26 (1.1) | 18 (0.8) | 17 (0.8) |
| *20-27 weeks* | 8 (<0.5) | 11 (0.6) | 13 (0.7) | 12 (0.6) | 11 (0.5) | 21 (0.9) | 15 (0.7) | 12 (0.5) | 16 (0.7) | 24 (1.1) |
